# Proteostasis and ageing dissidence

**DOI:** 10.1098/rstb.2025.0266

**Published:** 2026-10-01

**Authors:** Neethu Babu, Katie Whalen, Brian Freeman

**Affiliations:** Cell and Developmental Biology, University of Illinois at Urbana-Champaign, Urbana, IL 61801, USA

**Keywords:** proteostasis, molecular chaperone, ubiquitin–proteasome system, autophagy–lysosome pathway, ageing

## Abstract

Proteostasis, the process governing the dynamic regulation of protein synthesis, folding and degradation, is critical for maintaining cell function and organismal health. Ageing disrupts this intricate system, leading to inactive, misfolded and/or aggregated proteins that contribute to age-associated pathologies. Here, we explore current findings on proteostasis and its deterioration during ageing with an attention to three major pathways: molecular chaperone (MC), ubiquitin–proteasome system (UPS) and autophagy–lysosomal pathway. Components in all three paths undergo age-dependent decline in both expression and function, with each impairment having select initial outcomes on the health of a proteome. For example, loss in the MC path may cause nascent chain defects, whereas a decline in the UPS may result in the accumulation of toxic aggregates. Notably, the interconnectedness of these pathways results in reciprocal reactions in all three. Understanding how each path connects to the others and how these connections are regulated offers promising strategies to restore proteome integrity and extend a healthy lifespan.

This article is part of the Theo Murphy meeting issue ‘ProteostaSys: a systems view of proteostasis'.

## 1. Introduction

Homeostasis relies on balanced protein environments to accomplish a litany of tasks to create and maintain cells and organisms. The foundational mission of sustaining the lifecycle of all proteins is tightly controlled by the protein homeostasis (proteostasis) process as it governs the production of nascent polypeptide chains, activity of mature proteins/protein complexes and disposal of faltering or worn factors [1]. Notably, breakdown in proteostasis correlates with numerous ailments, including neurodegeneration, type II diabetes, cancers and heart disease [2]. At the core of the proteostasis network are the abundant molecular chaperones (e.g. Hsp70, Hsp90, T-complex protein Ring Complex (TRiC) and small heat shock proteins (sHSPs)) and proteolysis-related proteins (ubiquitin–proteasome and autophagy–lysosomal) [1]. Textbook models pitch specific roles for each factor within the proteostasis network, yet the system must readily evolve as the physiological status of a cell or organism changes. Hence, proteostasis must be a highly dynamic and reactive process that alters its flow in accordance with the needs of the protein population [3,4]. Here, we will discuss three fundamental and interconnected pathways enumerating proteostasis (molecular chaperone (MC), ubiquitin–proteasome system (UPS) and autophagy–lysosomal pathway (ALP)) and we will explore how the progressive decline of the MC pathway leads to the overwhelming of disaggregation and degradation machinery. While we will not delve deeply into the intricacies of each pathway, we encourage the reader to seek out more focused reviews detailing these fascinating paths [1,5–8].

Prototypical models portray defined roles for the individual factors working within each proteostasis pathway. For instance, during polypeptide biogenesis, Hsp70 and the nascent polypeptide-associated complex (NAC) are cast as the primary nascent chain binding factors that protect new proteins from aggregating as they emerge from a translating ribosome [9] (figure 1). Hsp70 then hands off the elongated nascent chains to other chaperones, including TRiC and Hsp90, where more intricate folding topologies can be mediated, metastable forms maintained and/or subunits assembled into large biostructures [10,11] (figure 1). Despite the appeal of this rudimentary paradigm, the fate of every nascent chain is likely idiosyncratic, being guided by the folding intermediates encountered during maturation, which, in turn, influence which other chaperones or co-chaperones are engaged to circumvent these forms, and all of which is influenced by the physiological status of the cell. Ominously, if a protein slips into a state recalcitrant to folding, it can contribute to pathogenic events that terminate in protein aggregates and plaques. Fortunately, other proteostasis components such as sHSPs and disaggregases can limit these toxic species by fostering soluble conformations or unfolding these tangled species and allowing the polypeptides to re-enter a folding path [12,13] (figure 1). Furthermore, post-translational modifications (e.g. ubiquitination) also influence protein solubility, activity and, if the polypeptide cannot be rescued, target it for degradation [14,15] (figure 1). Of note, the controlled degradation of erroneous polypeptides minimally recycles base materials and prevents detrimental interactions between inactive factors. On the whole, the proteostasis process uses a highly interconnected series of regulatory pathways to monitor, maintain and ensure proteome quality, thereby preventing the formation and accumulation of aggregation-prone, labile polypeptides.

**Figure 1.**
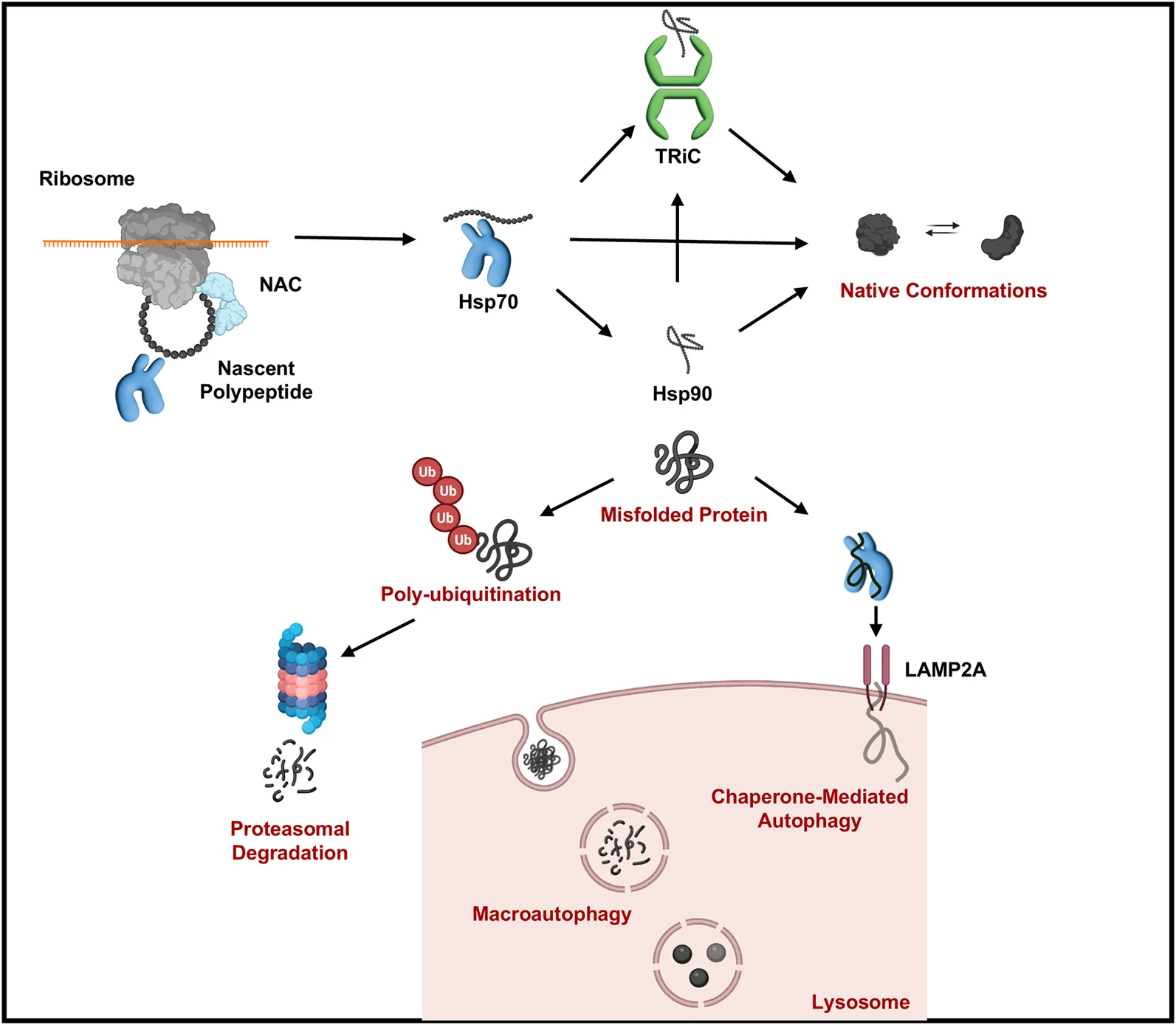
The proteostasis process is built on the molecular chaperone, ubiquitin–proteasome and autophagy–lysosomal pathways. Nascent polypeptides emerging from a ribosome are bound and protected by the combined actions of NAC and Hsp70. Client proteins requiring further assistance (folding, maintenance and/or assembly into larger structures) will be transferred to additional chaperones, including Hsp90 and TRiC. Proteins recalcitrant to reaching a native state (i.e. initial folding or refolding after damage) will be delivered to disposal machinery managed by either the ubiquitin–proteasome system (UPS) or autophagy–lysosomal pathway (ALP). Failure to remove labile, non-native polypeptides (i.e. unfolded or damaged proteins) leaves a cell vulnerable to a fitness reduction.

Unfortunately, the proteostasis system progressively breaks down during cellular and organismal ageing, which is exemplified by the accumulation of protein aggregates and/or plaques in aged cells and tissues [16]. While neurodegenerative ailments, including Alzheimer's and Parkinson's, exemplify proteostasis-related diseases, other catastrophic maladies, including type II diabetes and heart disease, are also triggered by proteostasis dysregulation [17–24]. Hence, it is imperative to better understand and further delineate the connection between ageing and proteostasis to better support human health. For instance, *how* and *why* does the proteostasis network collapse with age? While the accumulation of protein aggregates can demarcate aged cells, how did the cell attempt to adjust to the build-up of protein species destined for aggregation, and why did these safeguards fail? Is the decline of proteostasis triggered by age-related changes to the expression of certain chaperones or proteolysis proteins, or is it a functional decline in the proteostasis machinery, or an antagonistic combination? Here, we will address these outstanding questions and describe key events correlating with the age-associated collapse of proteostasis.

## 2. Ageing and protein synthesis decline: accuracy or survival?

From the moment a protein is synthesized to its eventual degradation, a protein's fate is monitored by the proteostasis network to maintain cell health. While it is clear that proteostasis breaks down with age (e.g. aggregates accumulate), where might this elaborate system begin to falter during ageing? An obvious source is at the start of polypeptide biogenesis—translation (figure 2). Protein translation is one of the most energy-intensive tasks within a cell, consuming approximately 30% of a cell's energy [25]. In young cells, this substantial investment ensures the accurate and steady production of functional proteins. However, as cells age, the rate of protein synthesis declines by approximately 50% [26,27]. It has been proposed that this significant reduction reflects a strategic reallocation of energy to deal with the burden of damaged or aggregated proteins [28,29]. Intriguingly, the decline in translation appears to be beneficial, as attenuating protein synthesis correlates with extended organismal lifespan, suggesting that the suppression of translation is not just a reaction to ageing but is a natural mechanism for promoting longevity [28,30].

**Figure 2.**
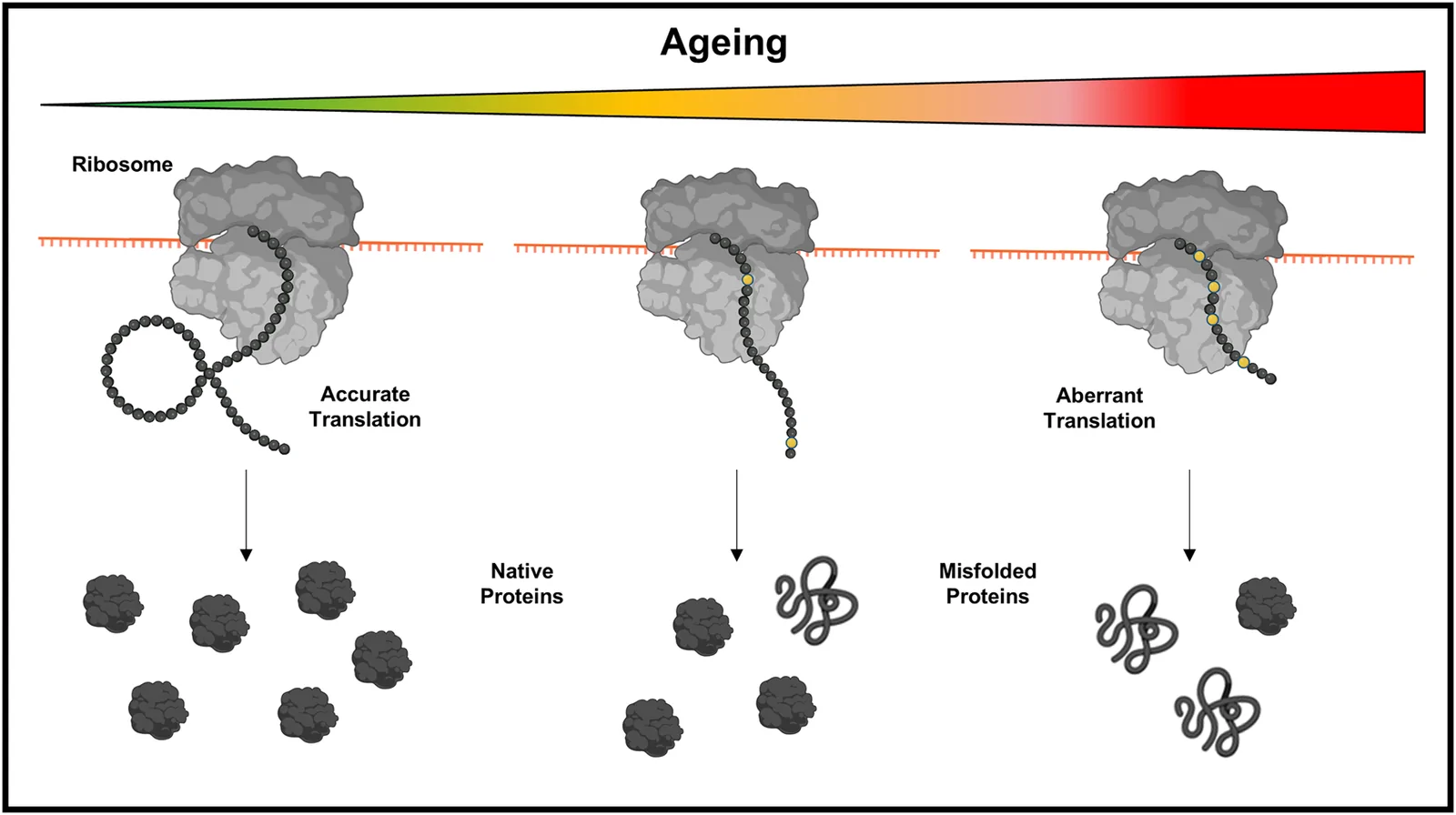
Protein translation is progressively more error-prone with age. The rate of protein translation decreases with age, and this decline is accompanied by an increase in translational error, such as incorrect amino acid incorporation (represented with yellow spheres). Mistranslation can interfere with proper protein folding, thereby leading to the accumulation of misfolded proteins.

The decline in protein synthesis with age minimally involves changes in the translational machinery. During natural ageing, ribosomal RNAs become imbalanced and free ribosomal protein subunits accumulate in the cytoplasm of older tissues [26,31]. In addition to machinery changes, the expression of regulatory proteins, including the central initiation factor eIF-2, declines proportionally with the translation rate during ageing [32]. Intriguingly, experimentally reducing ribosome levels by deleting genes encoding ribosomal proteins increases lifespan in both yeast and worms [30,33–35]. Similar lifespan extension has been achieved by suppressing certain translation initiation factors by RNA interference (RNAi) in worms [28,36–38]. Thus, the levels of translation components can serve to modulate ageing, and manipulating these factors is a viable strategy to alter lifespan.

In addition to the physical levels of translation factors, the activity and efficiency of the machinery are progressively compromised during ageing [39,40]. For example, translation accuracy declines with age as older cells have an increased occurrence of polypeptides with incorrect amino acids [41]. Importantly, translation of a single missense-containing protein is sufficient to hasten proteostasis collapse and organismal ageing, signifying the importance of accurate protein translation [42]. Recent ribosome profiling experiments have shown that translational errors correlate with altered translation initiation and ribosome pausing. In aged mouse models, there is a clear decline in initiation, as ribosome levels are depleted at the 5′ end of mRNA transcripts [43]. Even after initiating, other difficulties are encountered with age since ribosomes are more prone to pausing and collision [44,45]. In non-aged cells, the ribosome-associated quality control (RQC) pathway detects stalled ribosomes and degrades the incomplete polypeptides, but in aged cells RQC appears overwhelmed, leading to the accumulation of truncated polypeptides [44].

Translational errors place additional stress on an already burdened aged proteostasis network and correspond with reduced lifespan and increased disease susceptibility [46–49]. In older mice, diminished translational fidelity is associated with cognitive decline, including Alzheimer's-like symptoms and increased muscle tissue damage [50,51]. The heightened vulnerability of brain and skeletal muscle in age-related diseases is possibly explained by the substantial increase in translational errors within these tissue types [52]. Conversely, experimental models that enhance translational accuracy have prolonged lifespans, including in yeast, worms and flies [53]. Hence, accurate protein translation is vital for organismal health and longevity.

Besides altering the translation apparatus, a decline in polypeptide biogenesis can be triggered by changes in factors that protect nascent polypeptides as they leave the ribosome. For instance, the nascent polypeptide-associated complex (NAC), which complexes with ribosome to protect nascent chains [54,55], also has ribosome-independent functions. NAC functions as a molecular chaperone to prevent the aggregation of pathogenic polyQ proteins, and NAC overexpression delays the age-dependent paralysis of worms expressing polyQ proteins [54,56]. NAC may also serve as a general chaperone since knocking down NAC subunits leaves worms more vulnerable to heat shock [54]. Hence, as protein aggregation increases with age, NAC's ability to protect nascent chains may decline, thereby contributing to a faltering proteostasis system.

## 3. Declining chaperone capacity in ageing: the cost of misfolding

In actively dividing cells, tens of thousands of ribosomes can be translating polypeptides simultaneously, with the resulting nascent chains collectively imposing a substantial demand on proteostasis [57,58]. Fortunately, the molecular chaperone network has been effectively adapted to shepherd these labile polypeptides from harm within the cellular environment (figure 1). Besides NAC, Hsp70 plays an important role in nascent chain protection across all kingdoms of life [9], though, in bacteria, emerging nascent chains are first bound by trigger factor, a ribosome-associated chaperone that transiently shields hydrophobic patches before a hand-off to Hsp70 [59,60]. Hsp70 functions through a dynamic ATP-associated cycle that enables transient binding to hydrophobic polypeptide motifs, thereby allowing the chaperone to protect a client without interfering with its innate folding regime. The Hsp70–client binding cycle is modulated by the Hsp40 co-chaperones and nucleotide-exchange factors (NEFs) which help tailor substrate specificity and cofactor (client and ATP) turnover [61,62].

As cells age, Hsp70 activity declines [63]. This reduction has been attributed to age-associated oxidative modifications and diminished ATP availability, which compromise the chaperone's refolding cycle [64,65]. Likely, the Hsp70 inactivity contributes to the accumulation of toxic protein oligomers associated with neurodegenerative diseases [66,67]. Significantly, a sustained forced expression of Hsp70 extends lifespan and maintains proteostasis [68]. Intriguingly, the localized administration of exogenous Hsp70 by intranasal administration not only extends the lifespan of mice, but also improves memory [69]. Hence, Hsp70 is a proteostatic regulator of lifespan and a potential target for therapeutic intervention in the ageing process.

Besides activity, the overall protein levels of Hsp70 wane during ageing [70]. The age-associated decline in the DNA-binding activity of the main transcriptional regulator of chaperones, HSF1, likely contributes to the reduced expression of Hsp70s, as well as other molecular chaperones [71]. Studies in worms, flies and mammalian tissues indicate that HSF1 activity declines with age, blunting the heat shock response and disrupting proteostasis [72–74]. In yeast, HSF1 is essential for basal chaperone expression, but restoring the Hsp70 and Hsp90 chaperones alone is sufficient to support growth even in the absence of HSF1 [75]. Beyond yeast, HSF1 also has developmental roles in *Drosophila*, regulating larval growth and morphogenesis [76], and is frequently co-opted in cancers to sustain protein homeostasis in highly proliferative cells [77,78]. Chronic or constitutive activation of HSF1 enhances proteostasis and stress resistance and can extend lifespan in worms. However, it broadly reprogrammes the transcriptome, affecting genes involved in metabolism, development and reproduction, suggesting potential trade-offs that have not yet been experimentally observed [79,80]. These diverse roles underscore that HSF1′s contributions to ageing are likely complex, extending well beyond its canonical function in supporting chaperone expression [81,82].

The contribution of the chaperone network to proteostasis does not end with Hsp70, as the network continues to flow downstream. For some polypeptides, Hsp70 and its co-chaperones might be sufficient for folding *in vivo*, yet others require further assistance. The additional employed chaperones are likely dictated by the needs of the client itself. One potential route involves a hand-off to Hsp90, especially for metastable proteins, such as steroid hormone aporeceptors and unactivated kinases [83–85]. As the transfer involves a non-native, labile polypeptide, the chaperones must be in close proximity to maintain the client in a foldable state. For Hsp70 and Hsp90, there are at least two mechanisms: (i) a direct interaction between Hsp70 and Hsp90 that is conserved from bacteria to eukaryotes; and (ii) the eukaryotic tetratricopeptide repeat (TPR) co-chaperone HOP (Hsp70/Hsp90 organizing protein), which binds to the terminal ‘EEVD’ motifs of Hsp70 and Hsp90, and stabilizes the co-complex [86,87]. Why an additional mode arose in eukaryotes is not fully understood. It has been suggested that HOP might direct a shift in the pathway from folding to degradation for at least some clients [88]. Yet, other clients require HOP to reach a native, functional state [89]. Minimally, the two modes provide flexibility for the chaperone network that likely allows the system to react differently to the physical state of a client.

Once a client has engaged with Hsp90, its conformation can be manipulated. Hsp90 functions as a homodimer, cycling through multiple ATP-dependent conformations using its amino-, middle- and carboxyl-domains. In the absence of nucleotide, Hsp90 adopts an open V-shape tethered through its carboxyl-terminus. Upon ATP binding, the dimer shifts to a closed state at the amino-termini [90]. Initially, it was believed that Hsp90 only interacts with clients via a cleft formed between the two middle domains, and this may be the case for metastable clients [91,92]. For numerous other clients, binding can be mediated across the surfaces of all three Hsp90 domains by associating with intrinsically disordered regions of clients [93].

Although Hsp90 is traditionally described as a mediator of late-stage protein folding [94], our understanding of Hsp90 is evolving. Early biochemical studies showed that Hsp90 can suppress the aggregation of an unfolded model client (citrate synthase) and delay its heat-induced inactivation [94]. Further work showed that Hsp90 maintains non-native proteins in a folding-competent state *in vitro*, which became known as holdase activity [95]. *In vivo*, Hsp90 likely uses this function to protect or modulate the physical state of a client. By protecting a protein, Hsp90 keeps the client in a state that can enter downstream biological events, including activation by a small molecule (e.g. steroid aporeceptors) or import into an organelle (e.g. inner mitochondrial proteins) [96,97]. Alternatively, if Hsp90 binding is used to manipulate the conformation of a protein, this may allow the client to transition through energetically unfavourable physical states (e.g. release a DNA-bound transcription factor), thereby fostering the forward flow of other biological pathways (e.g. gene regulation) [98]. The holdase activity of Hsp90 extends to a wide variety of client proteins, including the earliest discovered substrates (i.e. glucocorticoid receptor, casein kinase II and oncogenic tyrosine kinase pp60v-Src) and up to approximately 20% of a cell's proteome [83–85,93]. To handle this extensive workload, Hsp90 itself comprises approximately 2% of total cellular protein mass [99].

Significantly, many of the known Hsp90 clients are linked to human disease, including factors contributing to neurodegeneration [100,101]. Hsp90 interacts with the intrinsically disordered Tau protein, modulating its ability to bind microtubules and suppressing its aggregation [102]. Hsp90 also inhibits the aggregation of *α*-synuclein, a pathogenic protein involved in Parkinson's disease, and reduces the toxicity of its oligomeric forms [103]. These findings emphasize that Hsp90 is a regulator of proteotoxicity and contributes to both healthy and pathogenic conditions. Like Hsp70, Hsp90′s activity also deteriorates with age. In Alzheimer's disease, Hsp90 can be sequestered by hyperphosphorylated Tau, thereby reducing its availability for other cellular roles and diminishing the cell's ability to manage stress and maintain proteome health [104]. In addition, ageing affects organelle-specific Hsp90 isoforms, suggesting a system-wide decline in Hsp90 surveillance [105,106]. Intriguingly, a single point mutation in the ATPase binding domain of Hsp90 recapitulates the lifespan extension typically observed under dietary restriction, as the cells have shifted to a protective, low-proliferative state in an attempt to maintain proteostasis [107,108]. Hence, dysregulation of proteostasis can occur at multiple points within the chaperone network.

Another key class of molecular chaperones is the chaperonins, which are large, multi-subunit, ring-shaped complexes that minimally offer a specialized environment for protein folding [109]. These assemblies provide a haven for vulnerable substrates by creating an enclosed chamber where folding can occur without interference from the crowded and reactive cellular environment. The best-studied example is the bacterial GroEL/ES system, which sequesters unfolded or misfolded proteins within the central GroEL cavity. GroES caps this chamber, allowing substrates to fold in isolation, shielded from interactions that may lead to misfolding and aggregation [110,111]. In eukaryotic cells, the cytoplasmic/nucleoplasmic chaperonin is the TRiC or CCT complex [112]. TRiC/CCT is particularly important for the folding of the essential, aggregation-prone actin and tubulin proteins [113,114]. These cytoskeletal proteins are delivered to TRiC/CCT not by Hsp70 but rather by a more specialized upstream chaperone, prefoldin, which is also known as GimC [115]. Interestingly, TRiC/CCT associates with approximately 10% of proteome, and a section of these polypeptides will be delivered by Hsp70, including the tumour suppressor complex VHL-VBC [10,116]. Thus, there are at least two routes to deliver a substrate to a chaperonin. In addition to its folding role, TRiC/CCT can suppress amyloid fibril formation for huntingtin protein (HTT) and *α*-synuclein [117,118]. As with Hsp70 and Hsp90, TRiC activity also decreases with age, including its ability to fold the critical cytoskeletal polypeptides [119]. At least in Huntington's disease, HTT binding contributes to the decline, as HTT association reduces TRiC availability, blocking it from essential folding duties [120]. Thus, disease-linked proteins can hijack chaperonin function, tipping the balance of proteostasis towards collapse.

## 4. Aggregation, disaggregation and the balancing act of proteostasis

When folding systems fail and proteins aggregate, cells employ disaggregases to restore proteome integrity [121]. The best-studied disaggregase is yeast Hsp104 [122]. In cooperation with Hsp70 and Hsp40, Hsp104 efficiently re-solubilizes aggregated proteins, thereby fostering re-entry into a folding pathway (figure 3) [123]. Hsp104 acts through tightly regulated cycles of substrate engagement and protein unfolding, where the client is threaded through its central hexameric channel [124]. Recent work has expanded our understanding of Hsp104′s roles in ageing. In quiescent ageing yeast, Hsp104 safeguards translation factors (e.g. eIF2 complex) and protects the nuclear proteome by disaggregating proteins in this cell compartment [125]. Hsp104′s nuclear activity is not passive, as it requires a carboxyl-terminal TPR-binding motif and engagement with nuclear import machinery, demonstrating compartmentalized regulation [122,125]. Supporting this system, Hsp110 (Sse1/Sse2 in yeast) emerges as an active participant in disaggregation and a crucial nucleotide exchange factor for Hsp70 [126]. Hsp110 maintains a supply of ATP-bound Hsp70 and assists directly at sites of aggregation, remodelling substrates and enabling client translocation through Hsp104 [127]. Loss of Hsp110 disrupts Hsp104 recruitment and stalls disaggregation, even when Hsp104 is present [128]. This integrated triage system thus couples disaggregation with post-aggregate refolding, promoting not just clearance, but functional recovery. Unfortunately, larger eukaryotes including humans do not encode an Hsp104 homologue. Other chaperones, including small heat shock proteins (sHSPs), can cooperate with Hsp70 and Hsp40 to disaggregate certain polypeptides [129]. In addition, sHSPs play a general role in maintaining the solubility of non-native proteins, thereby extending the time frame for folding by preventing the formation of aggregates (figure 3) [130].

**Figure 3.**
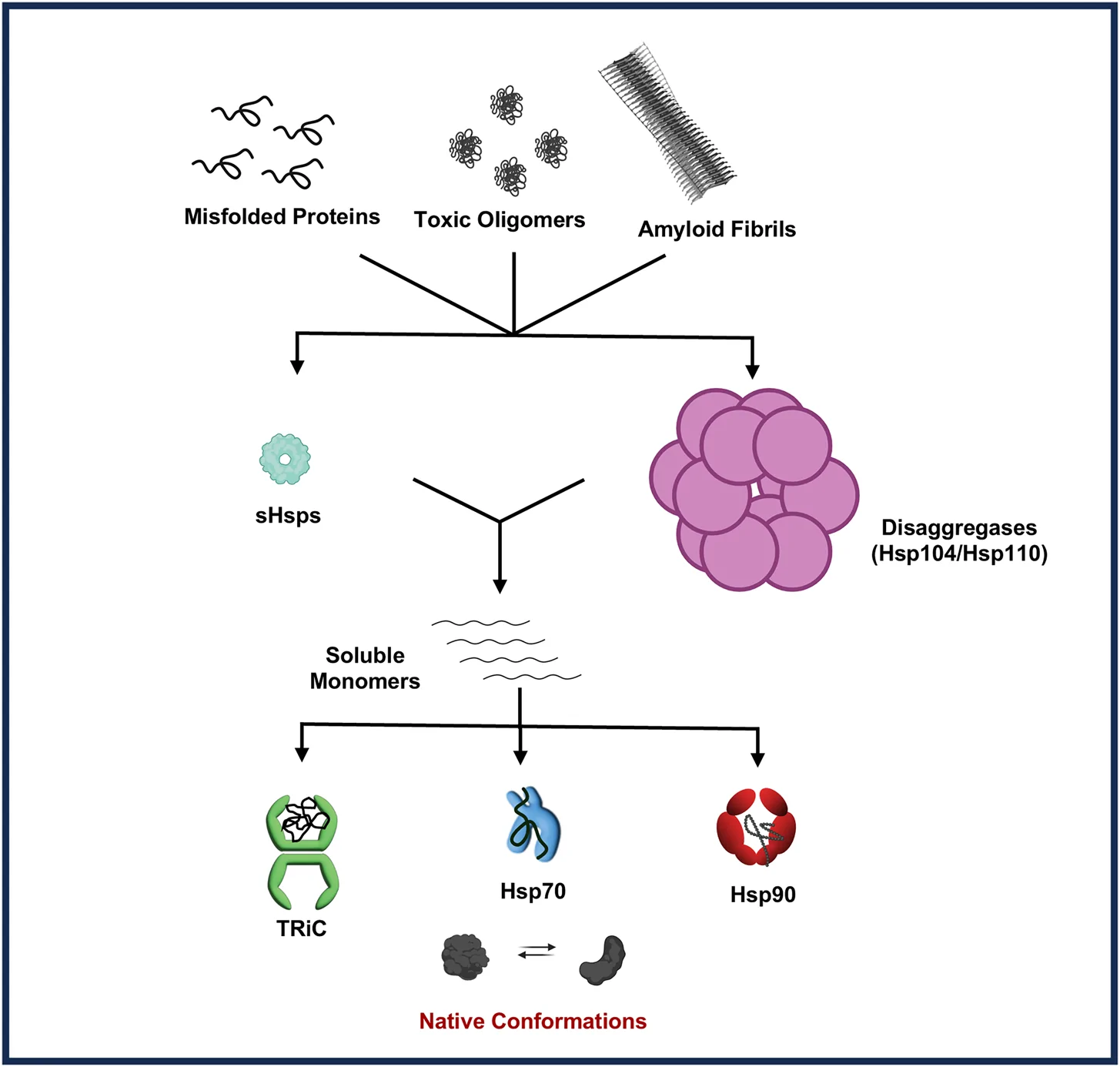
Molecular chaperones and protein disaggregases limit the accumulation of damaged, non-native proteins. Misfolded proteins can assemble into soluble oligomers or amyloid fibrils. Small heat shock proteins (sHSPs) and disaggregases such as Hsp104 and Hsp110 remodel these forms to create folding-competent protein species. These intermediates are processed by the molecular chaperone pathway, which includes Hsp70, Hsp90 and TRiC, to restore native structure and activity.

While generally viewed as pathogenic, protein aggregation can serve adaptive purposes. Soluble oligomers can activate protective responses, such as the HSF1-induction of chaperone levels [131]. In yeast, prion-like aggregates of Sup35, a translation termination factor that can adopt self-perpetuating amyloid conformations, have been linked to stress resilience [132]. Certain aggregases such as Btn2 and Hsp42 in yeast or HSP-17 in worms aid in the compartmentalization of misfolded proteins, preventing aberrant interactions [133]. These organized inclusion bodies stabilize misfolded proteins and may serve as temporary holding zones until the cell can re-engage folding or degradation pathways [134].

Likewise, structured aggregates like *α*-synuclein fibrils may function as sequestration sinks, reducing levels of more toxic intermediates [135], a notion that directly highlights a central controversy: if higher-order aggregates can be protective, what drives pathogenicity? Increasing evidence indicates that the most harmful species are the small, metastable oligomers that form before fibrils. For example, amyloid-beta (A β) dimers, which impair long-term potentiation (LTP) and memory, and compact 300–600 kDa prion protein (PrP) particles, which exhibit peak infectivity despite their modest size [136,137]. Accordingly, conditions that accelerate deposition into larger assemblies, such as reduced insulin-like growth factor-1 (IGF-1) signalling, can mitigate toxicity by forcing aggregation-prone proteins into hyperaggregated, less reactive states [138]. Thus, aggregation is not uniformly deleterious. Toxicity emerges primarily when proteins dwell in these transient oligomeric intermediates rather than when they are efficiently sequestered into inert, higher-order structures.

However, under conditions of ageing or chronic proteotoxic stress, the capacity of these sequestration mechanisms becomes increasingly burdened. During ageing, cytosolic and nuclear protein quality control systems, such as Juxtanuclear Quality Control (JUNQ), Intranuclear Quality Control (INQ) and Insoluble Protein Deposit (IPOD), show altered inclusions, increased aggregates and impaired clearance [133,139,140]. Declining chaperone and disaggregase capacity, trafficking defects and misfolded protein overload sequestration, allowing proteins to accumulate outside canonical quality control sites, highlighting how proteostasis failure drives age-related protein aggregation.

## 5. When the fail-safe fails: age-related protein degradation impairments

As polypeptides become irreversibly worn or broken, the proteostasis process must selectively remove these species from the intracellular space. The destruction or removal of polypeptides that otherwise cannot be saved (i.e. folded to a functional conformation) serves as the last resort to safeguard the native proteome within a cell. By and large, there are two main pathways for removal—proteolysis, with the proteasome acting as a workhorse degrading machine, and ALP, serving to remove large aggregates and even failed organelles [6]. Although both pathways are robust, each eventually succumbs to age, leaving the cell in a precarious position and ultimately resulting in death [141,142].

The UPS is tightly linked to molecular chaperones, which assist in substrate polyubiquitination and proteasomal targeting. The 19S particle of the 26S proteasome recognizes polyubiquitinated substrates, unfolds polypeptides and translocates chains into the 20S core, where they are cleaved [143]. Substrate polyubiquitination itself is controlled by a cascade of three enzyme types: E1 ubiquitin-activating enzymes, E2 ubiquitin-conjugating enzymes, and E3 ubiquitin ligases which selectively tag substrate proteins [144]. Catalysis by the proteasome is often associated with K48-linked polyubiquitination, where multiple ubiquitin molecules are covalently attached to lysine 48 [145], while K63-linked polyubiquitination was recently found to be used for the lysosomal degradation of certain proteins via microautophagy [146]. Thus, the polyubiquitination of different amino acids can lead to alternative protein fates.

There is an extensive network of interactions between chaperones and E3 ubiquitin ligases to mediate this specific polyubiquitination. These interactions are further moderated by co-chaperones and deubiquitylating enzymes, which provide additional layers of control [144], emphasizing the importance of tightly regulated protein degradation. Despite the selectivity of polyubiquitin tagging, many E3 ubiquitin ligases are non-essential, suggesting that these enzymes can work in parallel [144]. By contrast, several E3 ubiquitin ligases have location-specific effects, such as San1 in the nucleus and Rsp5 targeting of plasma membrane proteins [147,148]. Overall, interaction of molecular chaperones with diverse E3 ubiquitin ligases leads to reversible decisions about protein fate, where erroneous polypeptides are sentenced to degradation instead of refolding.

There are several known interactions between cytosolic E3 ubiquitin ligases and molecular chaperones, such as the association of the E3 ubiquitin ligase Ubr1 with Hsp70, Hsp40 and Hsp110 [144]. One of the most well studied UPS–MC linkages is the association of Hsp70 and Hsp90 with the CHIP cofactor [149,150]. CHIP functions as a dimer, having an amino-terminal TPR domain that binds to the carboxyl-terminal EEVD motifs of Hsp70 and Hsp90 and blocks the ATPase and folding cycles of the chaperones [151]. The carboxyl-terminus of CHIP has E3 ubiquitin ligase activity which can scaffold the chaperones with their bound clients to the UPS, including the stress-associated E2 ubiquitin-conjugating enzymes Ubc4/5 [149,152]. While the MC–UPS connection is often considered for worn polypeptides, these events can start during protein translation. Nascent ribosomal proteins can be sequestered into nucleolar bodies, polyubiquitinated and degraded by the proteasome in a chaperone-dependent manner [153]. Hence, the interconnection between the MC and UPS pathways dynamically reacts to the needs of the proteome.

Comparable to chaperones, the activity of the proteasome declines with age. Notably, it remains unclear whether this decrease in proteasome activity is a consequence of MC pathway failure or a correlative decline. The chymotrypsin-like proteasome activity drops to 55% in late-stage replicative senescence in fibroblasts, and 26S proteasome activity decreases by 50% in aged mouse brains [154,155]. These declined activities correlate with an accumulation of toxic proteins associated with disorders including Alzheimer's and Parkinson's [156–161]. Significantly, enhancing proteasome activity can accelerate the clearance of potentially toxic species, including amyloid precursor proteins [162]. The age-associated decrease in proteasome function is owing, in part, to imbalances in the expression of UPS components. For example, in ageing fibroblasts proteolytic activity declines in parallel to proteasome subunit expression, which correlates with shorter lifespan [158,163]. Organismal ageing also influences the stoichiometry of uncapped to capped proteasomes. In aged flies and vertebrates, the ratio of 20S to 26S proteasomes shifts along with the protein degradation capacity [163,164]. Conversely, boosting proteasome activity increases longevity [165].

In addition to proteasome units, ubiquitination enzymes are also dysregulated with age. For example, during cataract formation, which is caused by protein aggregates in the eye lens, E2 ubiquitin-conjugating enzymes decrease while E3 ubiquitin ligases increase [166,167], and these changes are consistent with altered ubiquitination patterns in aged lens [168]. Changes in ubiquitination enzymes also impact lifespan, as mutations in the ubiquitin fusion degradation complex and E1 ubiquitin-activating enzyme Uba1 reduce longevity, while overexpression of the E3 ubiquitin ligase parkin extends lifespan [169–171].

Significantly, the dysregulation of the UPS correlates with the progression of several age-related diseases, as highlighted by the accumulation of polyubiquitinated proteins in the brains of Alzheimer's patients [172]. The E2 ubiquitin-conjugating enzyme UBE2N has been implicated in the progression of amyotrophic lateral sclerosis (ALS), since UBE2N levels increase with age and promote the aggregation of the central ALS-associated protein TDP-43 [173]. Another E2 ubiquitin-conjugating enzyme, UBE2D, can facilitate the degradation of the pathogenic HTT protein. UBE2D knockdown leads to HTT aggregation and shortens lifespan in flies [174]. Hence, alterations in UPS components can influence events associated with proteopathies. Other post-translational modifications also influence HTT pathogenicity. The small ubiquitin-like modifier (SUMO) tag, which is thought to compete with ubiquitin to prevent degradation, has independent effects on protein solubility and can prevent HTT aggregation [175]. Additionally, HTT phosphorylation promotes degradation by both the proteasome and autophagy [176]. Thus, the age-associated dysregulation of post-translational modifications related to degradation contributes to the progression of proteopathies.

The autophagy–lysosomal pathway (ALP) is deeply intertwined with molecular chaperones, and its activity similarly decreases with age. It is well established that this loss of function is linked to multiple neurodegenerative conditions [177–182]. For example, the autophagy-related proteins (Atgs) Atg5, Atg7 and Atg8 all contribute to the inhibition of protein aggregate accumulation, slow the onset of age-associated phenotypes and increase lifespan [183–189]. Yet, the influence of Atgs in proteopathies is not always clear. For instance, in a mouse model for amyotrophic lateral sclerosis, it is the loss of Atg7 that extends lifespan [190]. Hence, there is still much to be understood about the connection between autophagy, ageing, proteopathies and the integrated roles of molecular chaperones.

Like the UPS, the ALP can be directly linked to molecular chaperones, which influence the function of ALP components. In macroautophagy, cellular contents are engulfed by a double-membrane autophagosome that fuses with a lysosome to degrade the material [191]. Interestingly, a key modulator of autophagy, the Atg1/Ulk1 kinase, is dependent upon Hsp90 for activity [192]. Hence, loss of Hsp90 will impair initiation of autophagy. In chaperone-mediated autophagy (CMA), Hsp70 recognizes clients containing a KFERQ motif and directs these to LAMP2A receptors, which channel the clients for lysosomal degradation [193]. In addition to mediating client flow, Hsp70 also modulates LAMP2A dynamics by prompting monomerization of LAMP2A, whereas Hsp90 stabilizes LAMP2A, including facilitating multimerization of the receptor [194]. CMA is particularly important for targeting disease-related proteins, including Tau and *α*-synuclein. In mouse models, deletion of LAMP2 correlates with Tau elevation and accelerated Alzheimer's pathology [195]. Likewise, CMA is impaired in Parkinson's disease, where *α*-synuclein accumulates in inclusion bodies [196,197]. Therefore, CMA fulfils an important role in regulating protein degradation, and CMA failure contributes to age-associated neurodegenerative disorders. Hence, the MC and ALP pathways share important connections for maintaining proteostasis.

## 6. Conclusions

The proteostasis process ensures that all proteins produced within a cell are properly synthesized, folded, trafficked, active, modified and eventually degraded. A dynamic balance between the MC, UPS and ALP pathways creates an active proteome which is critical to support cell function, enable healthy ageing and avoid disease states. Here, we have summarized key discoveries highlighting the connectedness of these pathways and how disjunction within and/or between them disrupts proteostasis, leading to the accumulation of protein aggregates and hastening cell dysfunction and death. Despite the wealth of information detailing proteostasis events, our understanding likely only scratches the surface of this complex process.

Building on the studies discussed in this review, a potential working model emerges in which age-dependent declines contribute to proteostasis collapse. These include chronic proteotoxic load together with reduced stress-inducible chaperone capacity, which is shaped in part by changes in nutrient availability, growth signalling pathways such as insulin/IGF-1 and TGF-β/activin, and stress-responsive transcriptional regulators such as HSF-1, whereas accumulation of protein aggregates, impaired autophagy–lysosome and proteasome activity, and organelle dysfunction are either downstream consequences or correlates of an already exhausted network. In this framework, these upstream signalling pathways tune the basal capacity and inducibility of the MC and the UPS/ALP systems, and thereby determine when cells cross a threshold from compensated stress to irreversible proteostasis failure. Over time, many stress-responsive mechanisms that initially help maintain proteostasis can become counterproductive. For example, short-term reductions in translation, increased protein sequestration or formation of aggregates may protect cells early on but lead to impaired recovery and accumulation of damage under chronic stress or during ageing. Highlighting this shift from adaptive to maladaptive outcomes emphasizes that proteostasis decline reflects a gradual loss of flexibility in the network rather than a single failure point. Explicitly distinguishing these putative drivers from their downstream consequences highlights testable hypotheses for future work, including whether late-life reinforcement of chaperone and autophagy pathways, or targeted modulation of signalling pathways, can restore proteostasis and delay age-associated functional decline.

## Data Availability

This article has no additional data.

## Theme

One contribution of 20 to a Theo Murphy meeting issue ‘ProteostaSys: a systems view of proteostasis'.
